# NVP-BEZ235, a dual PI3K-mTOR inhibitor, suppresses the growth of FaDu hypopharyngeal squamous cell carcinoma and has a synergistic effect with Cisplatin

**DOI:** 10.1038/s41420-018-0060-7

**Published:** 2018-05-10

**Authors:** Cheng-Ming Hsu, Pai-Mei Lin, Yao-Te Tsai, Ming-Shao Tsai, Chun-Han Tseng, Sheng-Fung Lin, Ming-Yu Yang

**Affiliations:** 1 Department of Otolaryngology, Chiayi Chang Gung Memorial Hospital and Chang Gung University College of Medicine, Chiayi, Taiwan; 2 School of Traditional Chinese Medicine, College of Medicine, Chang Gung University, Taoyuan, Taiwan; 3 Department of Nursing, I-Shou University, Kaohsiung, Taiwan; 4 Division of Hematology and Oncology, Department of Internal Medicine, E-Da Hospital, Kaohsiung, Taiwan; 5 School of Medicine, I-Shou University, Kaohsiung, Taiwan; 6 Graduate Institute of Clinical Medical Sciences, College of Medicine, Chang Gung University, Tao-Yuan, Taiwan; 7 Department of Otolaryngology, Kaohsiung Chang Gung Memorial Hospital and Chang Gung University College of Medicine, Kaohsiung, Taiwan

## Abstract

NVP-BEZ235 is a dual phosphoinositide 3-kinase (PI3K)-mammalian target of rapamycin (mTOR) inhibitor. A dual approach targeting more than one downstream effector is a promising strategy for treating cancers. The aim of this study was to evaluate the effect of NVP-BEZ235 in treating FaDu hypopharyngeal squamous cell carcinoma (HSCC), either alone or in combination with cisplatin. We found *mTOR* expression was higher in patients with HSCC. In the in vitro study, treatment with NVP-BEZ235 alone attenuated cell proliferation and suppressed p-p70S6K and p-4E-BP1 expression in FaDu cells. When NVP-BEZ235 was combined with Cisplatin, apoptosis was induced more effectively than with either drug alone. In mice with a FaDu xenograft, cotreatment with NVP-BEZ235 and Cisplatin engendered synergistic effects and produced a greater antitumor response than did treatment with either drug alone. Resected tumor samples also showed decreased p-p70S6K expression. Collectively, these data demonstrate that NVP-BEZ235 inhibits HSCC growth through phospho-p70S6K suppression and has a synergistic effect with Cisplatin in treating HSCC. The data also provide a strategy for more effective HSCC treatment.

## Introduction

Head and neck squamous cell carcinoma (HNSCC) is the sixth most common cancer around the world. HNSCC affects nearly 600,000 new patients every year and its mortality rate is approximately 50%. Hypopharyngeal squamous cell carcinoma (HSCC), a less prevalent cancer arising from the mucosa of the upper aerodigestive tract, accounts for 3–5% of all HNSCC cases^1^. Most patients with HSCC are diagnosed only in the advanced stage. In a large series study^2^, approximately 70–85% of the patients were at Stage III or IV at presentation, with 5-year overall survival rates of approximately 15–45%.

Platinum combination regimens are considered the standard first-line treatment for patients with inoperable, recurrent, or metastatic HNSCC^3,4^. However, if *cis*-diamminedichloridoplatinum (CDDP)-based chemotherapy fails, these patients must receive salvage total laryngectomy. If they were still inoperable, only limited therapeutic options are available and most could receive only palliative radiation or supportive care^5–7^. Treatment failure is mostly due to the development of CDDP resistance, which compromises the treatment outcome and patient survival. Therefore, novel agents that can significantly enhance the effects of existing chemotherapeutic drugs with less toxicity are needed for possible organ preservation.

With progress in cancer research, targeted therapy is becoming a first- or second-line treatment option for various malignant diseases including HSCC^8^. Many preclinical studies have demonstrated synergistic antitumor effects when combining targeted therapy with CDDP^9,10^. The phosphoinositide 3-kinase (PI3K)-protein kinase B (Akt)-mammalian target of rapamycin (mTOR) intracellular signaling pathway is influential in various physiological activities, including cellular survival, proliferation, migration and differentiation, as well as in protein synthesis, angiogenesis, and glucose metabolism. In addition, the PI3K–Akt–mTOR pathway is associated with many oncogenic processes and is one of the most frequently dysregulated signaling pathways in cancer, including hypopharyngeal cancer^11^. The PI3K-Akt-mTOR pathway provides unique opportunities for anticancer therapy, because it is often constitutively activated in human cancer cells. Therefore, targeting of PI3K-Akt-mTOR signaling could be a reasonable strategy in the treatment of hypopharyngeal cancer where systemic therapy is effective, especially in advanced disease. NVP-BEZ235 is an imidazo[4,5-c]quinoline derivative that inhibits PI3K and mTOR kinase activity by binding to the adenosine triphosphate-binding cleft of these enzymes^5^. NVP-BEZ235 is a dual PI3K-mTOR inhibitor. Because targeting more than one downstream effectors may delay or even prevent therapy resistance, the dual approach is promising^12^. NVP-BEZ235 exhibited antitumor activity in lung cancer^13,14^, cells of human glioma^15,16^, breast cancer^17,18^, melanoma^19^, pancreatic cancer^20,21^, sarcoma^12,22^, nasopharyngeal cancer^23,24^, and hepatoma^25–27^. NVP-BEZ235 as a PI3K-mTOR inhibitor is currently in Phase I/II clinical trials and has shown great promise in treating solid tumors in preclinical mouse models^15^.

In this study, we demonstrated the effect of NVP-BEZ235 on PI3K-Akt-mTOR signaling in FaDu cells both in vitro and in vivo. Furthermore, we demonstrated that Cisplatin and NVP-BEZ235 synergistically inhibited the proliferation of FaDu cell lines with an enhanced reduction of mTOR signaling and p70S6K levels. Cisplatin and NVP-BEZ235 treatment also synergistically inhibited FaDu cells in vivo by exhibiting a significant reduction in tumor burden and p-p70S6K expression after treatment.

## Results

### Analysis of mTOR expression in patients with HSCC by using real-time quantitative reverse transcriptase-polymerase chain reaction

**Fig. 1:**
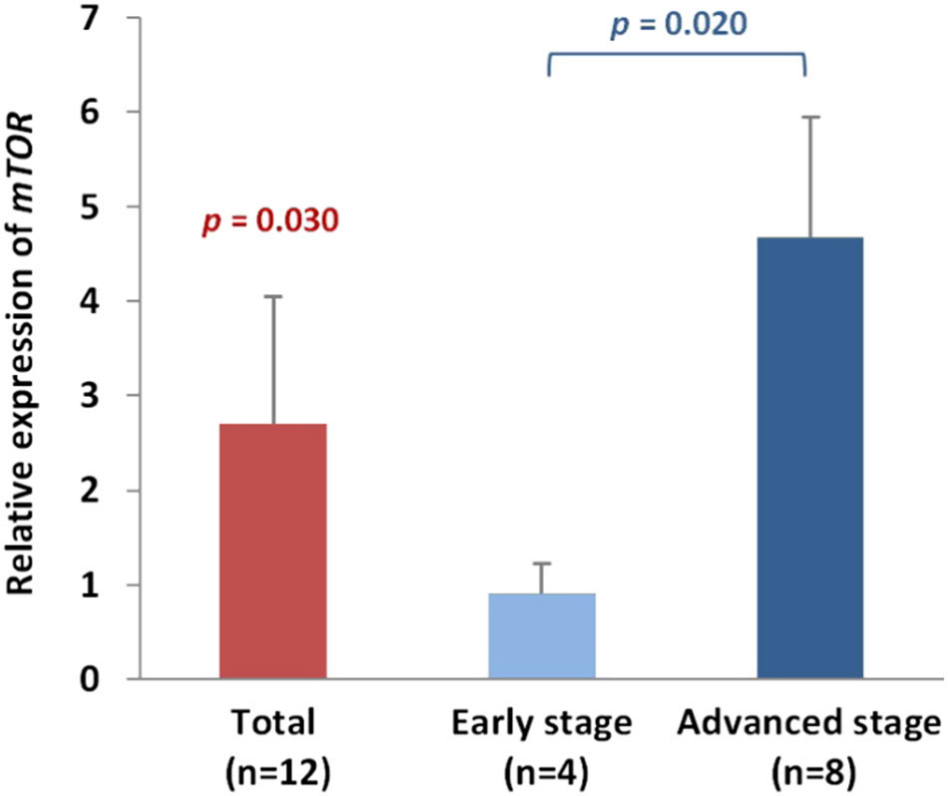
*mTOR* expression in HSCC. *mTOR* expression was upregulated in cancerous tissues of HSCC (*p* = 0.030) and was higher in advanced hypopharyngeal cancerous tissues compared with early hypopharyngeal cancerous tissues (*p* = 0.020). The y-axis represents the fold change in the *mTOR* expression level of cancerous tissues relative to noncancerous parts. The relative expression level in cancerous tissues was calculated by the comparative C*t* (ΔΔC*t* method). The mean *mTOR* expression level in noncancerous parts was assigned a value of 1, whereas the *mTOR* expression level in cancerous tissues was calibrated to obtain the fold change in cancerous tissues

Cancerous and noncancerous tissues from 12 patients with HSCC were examined for the expression of *mTOR* by using real-time quantitative reverse transcriptase-polymerase chain reaction (qRT-PCR) in order to elucidate whether the expression levels of mTOR were altered in cancerous tissues. Our data revealed that the *mTOR* expression levels were significantly upregulated in hypopharyngeal cancer (*p* = 0.030). Specifically, *mTOR* levels in advanced hypopharyngeal cancerous tissues showed a 5.15-fold increase compared with those in early hypopharyngeal cancerous tissues (*p* = 0.020) (Fig. 1).

### NVP-BEZ235 downregulated PI3K-Akt-mTOR signaling pathway in FaDu HSCC cells: suppression of p-mTOR and p-p70S6K

**Fig. 2:**
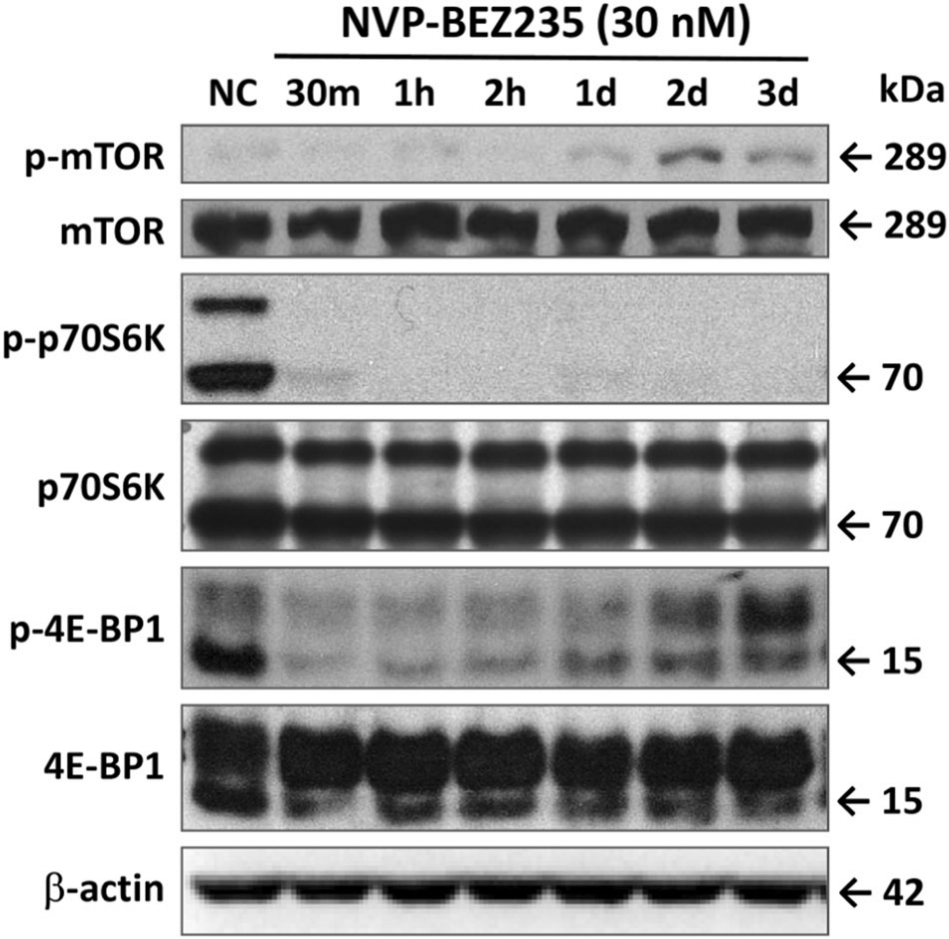
NVP-BEZ235 inhibited downstream targets of mTOR pathway. FaDu HSCC cells were cultured in six-well plates and treated with 30 nM NVP-BEZ235 for 30 min (30 m), 1 h (1h), 2 h (2h), 1 day (1d), 2 days (2d), and 3 days (3d). Protein extracts of cells were analyzed by Western blotting with antibodies against p-mTOR, mTOR, phospho-p70S6K (p-p70S6K), p70S6K, phospho-4E-BP1 (p-4E-BP1), 4E-PB1, and β-actin

We investigated whether NVP-BEZ235 could inhibit the phosphorylation of downstream targets of the mTOR pathway. In NVP-BEZ235-treated FaDu cells, mTOR, p70S6K, and 4E-BP1 phosphorylation was effectively reduced within 30 min, but the corresponding total proteins were not affected (Fig. 2). The phospho-p70S6K level was completely suppressed for 3 days; the phospho-mTOR (p-mTOR) and phospho-4E-BP1 levels were suppressed for 1 day.

### NVP-BEZ235 and Cisplatin combination synergistically inhibited proliferation of FaDu HSCC cells and induced cell apoptosis

The median-effect principle is often used to analyze concentration–response data for a single drug or a combination of drugs. In this principle, the potency (*D*_*m*_ or half maximal inhibitory concentration [IC_50_]) and the shape of the concentration–effect curve (*m* value) are considered. However, to analyze whether NVP-BEZ235 has a synergistic effect, IC_20_ (20% inhibitory concentration) values were determined to choose a constant Cisplatin concentration ratio.

**Fig. 3:**
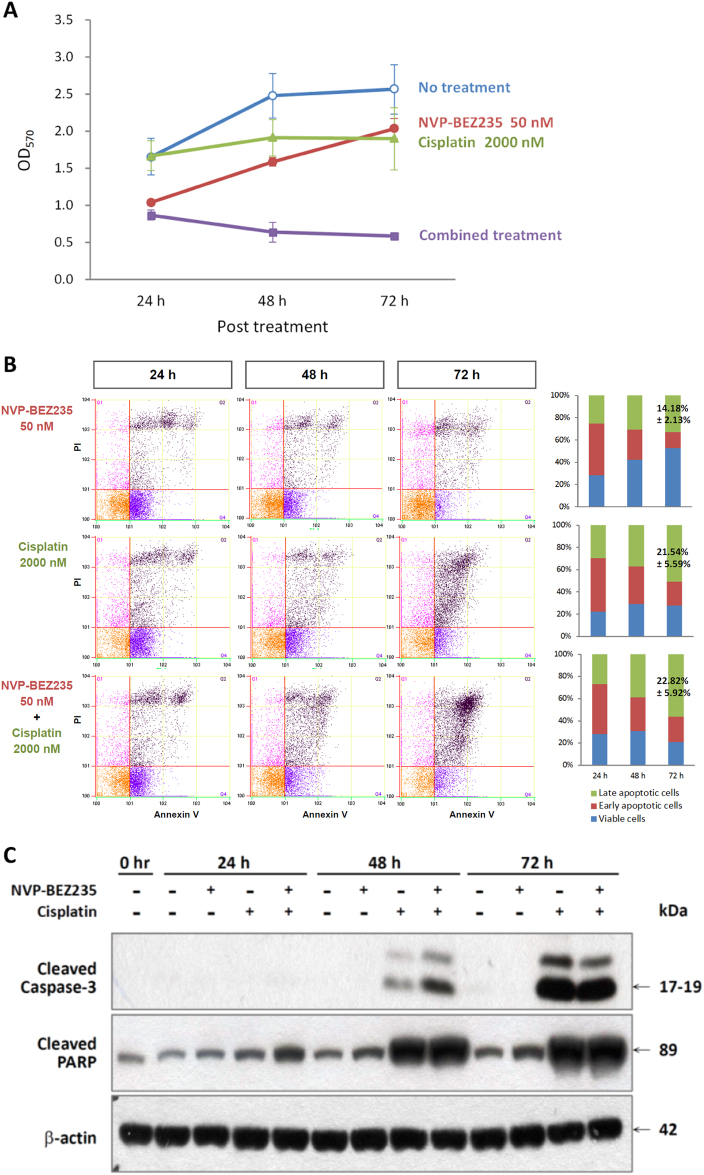
NVP-BEZ235 and Cisplatin synergistically inhibited cell proliferation and induced apoptosis of FaDu HSCC cells. **a** Inhibitory effects of NVP-BEZ235, Cisplatin, or a combination of NVP-BEZ235 and Cisplatin on FaDu HSCC cells were assessed by MTT methods after 24, 48, and 72 h of treatment. Data presented are the mean and SE of three independent experiments. **b** Combination of NVP-BEZ235 and Cisplatin synergistically induced apoptosis. FaDu HSCC cells were treated with NVP-BEZ235, Cisplatin, or a combination of NVP-BEZ235 and Cisplatin for 72 h; the percentages of apoptotic cells were determined by Annexin V/PI staining followed by flow cytometric analysis. **c** Combination of NVP-BEZ235 and Cisplatin synergistically induced cleavages of Caspase 3 and PARP, as determined by Western blotting

We assessed the antiproliferative potential of Cisplatin and NVP-BEZ235 on FaDu cells. We performed a 3-(4,5-dimethylthiazol-2-yl)-2,5-diphenyltetrazolium bromide (MTT) assay and manually counted viable cells to determine the effects of NVP-BEZ235 on cell growth. After 72 h of treatment, the combined treatment with NVP-BEZ235 and Cisplatin had significantly inhibited the growth of FaDu cell lines compared with the treatment with NVP-BEZ235 or Cisplatin alone (Fig. 3a).

The combined treatment of FaDu cells with NVP-BEZ235 and Cisplatin markedly increased the numbers of late apoptotic cells (22.82% ± 5.92%) compared with the untreated control (8.22% ± 1.26%) and treatment with Cisplatin (21.54% ± 5.59%) or NVP-BEZ235 (14.18% ± 2.43%) alone (*p* < 0.05; Fig. 3b), as demonstrated by flow cytometric analysis after Annexin V–Fluorescein isothiocyanate (FITC)/propidium iodide (PI) double staining. The combined treatment of FaDu cells with NVP-BEZ235 and Cisplatin also enhanced the cleavage of the apoptosis-related proteins caspase-3 and PARP. The cleavage of caspase-3 and PARP increased significantly at 48 and 24 h, respectively, compared with NVP-BEZ235 alone and Cisplatin alone (Fig. 3c). These data suggest that NVP-BEZ235 could sensitize Cisplatin-induced proliferation inhibition and apoptosis.

### Combination of NVP-BEZ235 and Cisplatin-induced cell cycle arrest in G2/M phase

**Fig. 4:**
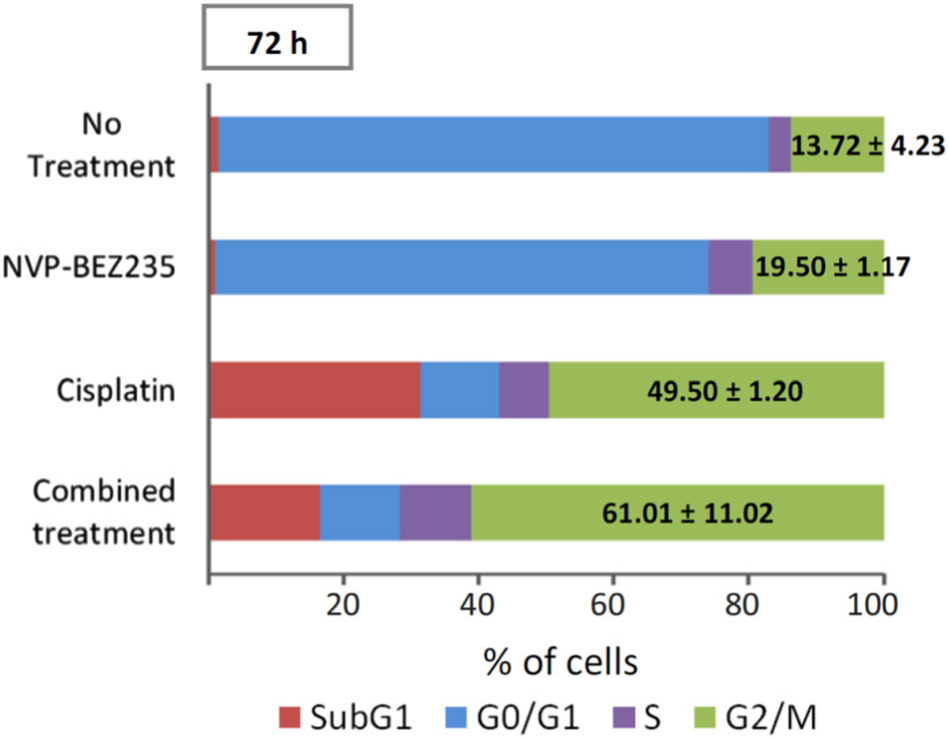
Effect of NVP-BEZ235 and Cisplatin on cell cycle progression. FaDu HSCC cells were treated with NVP-BEZ235, Cisplatin, or a combination of NVP-BEZ235 and Cisplatin for 72 h; the proportion of cells in each phase of the cell cycle was calculated as the percentage of the total cell population. Data presented are the means and SE of three independent experiments

To study the antiproliferative mechanism of NVP-BEZ235 in HSCC cells, we tested whether NVP-BEZ235 treatment affects the cell cycle. FaDu cells were cultured with 50 nM NVP-BEZ235, 2000 nM Cisplatin, or a combination of the two for 72 h, and cell cycle arrest was analyzed using flow cytometry. As shown in Fig. 4, the combined treatment resulted in a marked increase in cells in the G2/M phase (61.01% ± 11.02%) compared with NVP-BEZ235 (19.50% ± 1.17%) or Cisplatin (49.50% ± 1.20%) alone at 72 h. These results suggest that NVP-BEZ235 may enhance the cytostatic effect of Cisplatin by promoting G2/M phase accumulation and inhibiting cell cycle progression.

### NVP-BEZ235 showed synergistic antitumor activity with Cisplatin and suppressed phosphor-p70S6K in in vivo xenograft models

**Fig. 5:**
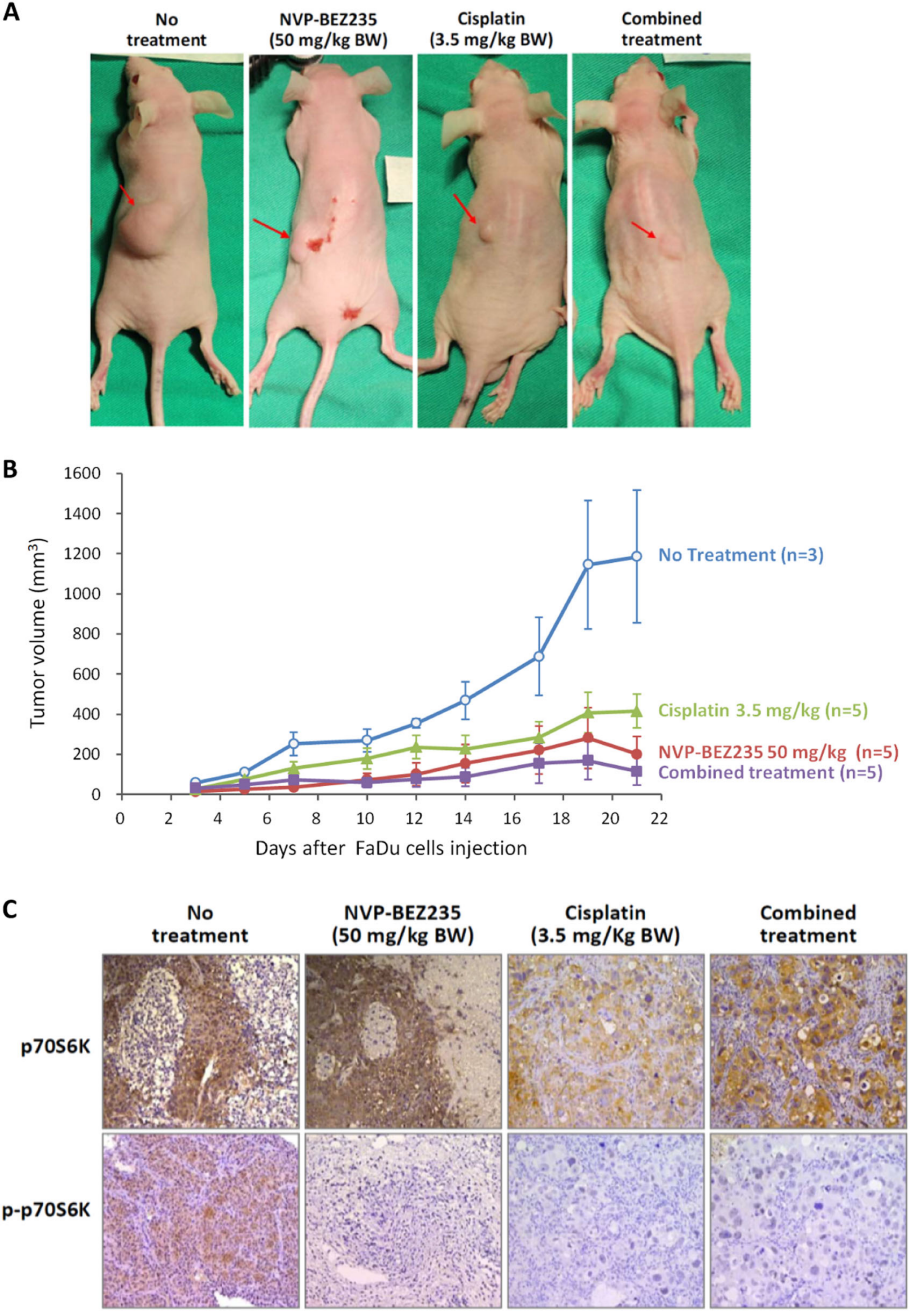
Antitumor effect of NVP-BEZ235 and its synergy with Cisplatin in FaDu HSCC xenograft. **a, b** Antitumor effects of NVP-BEZ235, Cisplatin, and a combination of NVP-BEZ235 and Cisplatin in FaDu tumor xenografts determined by calculating tumor volumes. **c** Immunohistochemical staining of p70S6K and p-p70S6K. Combination of NVP-BEZ235 and Cisplatin inhibited p-p70S6K expression in FaDu tumor xenografts

To examine the antitumor effects of NVP-BEZ235 in vivo, we established a nude mouse (BALB/cAnN.Cg) model by using the FaDu cell xenograft. Because the in vitro study showed that the effect of the combined treatment on cell apoptosis was superior to that of single treatment involving either Cisplatin or NVP-BEZ235 alone, we anticipated that the combined treatment would further suppress tumor growth in vivo. To confirm our assumptions, FaDu cells were injected subcutaneously into nude mice to establish xenograft tumors. The mice were then treated with NVP-BEZ235 (50 mg/kg) by oral gavage, Cisplatin (3.5 mg/kg) by intraperitoneal injection, or a combination of the two for 21 consecutive days. As shown in Fig. 5a, the combined treatment resulted in more significant tumor suppression compared with monotherapy. Quantitative analysis indicated that the tumor volume in the group subjected to the combined treatment was less than 50% of the volumes in the groups subjected to treatment with NVP-BEZ235 or Cisplatin alone. The results of the Western blot analysis from the xenograft tumor showed that the drug combination led to an enhanced reduction in phosphor-p70S6K (Fig. 5c).

## Discussion

This is the first study to investigate the effect of NVP-BEZ235 therapy on FaDu (HSCC) cells. NVP-BEZ235 is a novel orally available dual PI3K-mTOR inhibitor, which is currently used in Phase I/II clinical trials and was revealed to control solid tumors in preclinical mouse models^16^. The PI3K-Akt-mTOR signaling pathway and its abnormal activation were reported to be influential in the progression, metastasis, and chemoresistance of a variety of tumors^28^.

Our in vitro results demonstrated that NVP-BEZ235 could significantly suppress mTOR, 4EBP1, and p70S6K activities. Moreover, p-mTOR and phospho-4EBP1 were each suppressed in the initial 24 h, but phospho-p70S6K did not return to the normal level for 72 h. A previous study reported that phospho-p70S6K downregulated by targeted therapy may benefit patients through the inhibition of tumor growth as well as metastasis^29^. Our results also show that either NVP-BEZ235 alone or Cisplatin alone could inhibit cell proliferation and induce apoptosis, but the combination of NVP-BEZ235 and Cisplatin could synergistically induce FaDu cell cycle arrest in the G2/M phase and cell apoptosis more significantly, thereby reducing proliferation and survival more effectively. Cancer drugs exert antitumor effects through cell cycle arrest which is one of the important antitumor mechanisms. The mechanism underlying NVP-BEZ235-induced apoptosis of HSCC cells is not entirely clear; thus, additional in-depth studies should be conducted in this regard. Previous studies have reported that the S6 kinase (S6K) plays various roles in different mechanisms of apoptosis^30,31^. In the PI3K-Akt-mTOR pathway, mTOR activation results in the phosphorylation of numerous substrates, including the phosphorylation of S6K by the mammalian target of rapamycin complex 1. The effect of NVP-BEZ235 on apoptosis of FaDu cells is associated with the phosphorylation of S6K, which may play important roles in this effect. NVP-BEZ235 exhibits the antitumor effect not only by inhibiting Akt survival pathway but also by promoting cell apoptosis. These raise the possibility of using the combination treatment to develop a promising therapeutic strategy to enhance the effects of chemotherapy and improve clinical outcomes for patients with head and neck cancer.

We observed that mice fed oral NVP-BEZ235 may exhibit a reduction in body weight. The PI3K-Akt pathway promotes cell survival and inhibits apoptosis; moreover, the alteration of intracellular signaling through the PI3K-Akt pathway promotes proliferation and sustains a higher demand of transformed cells for metabolic input by upregulating glycolysis through the Warburg effect^32^. NVP-BEZ235 will thus have a potential to affect glucolysis of tumor and normal cells. The dose of NVP-BEZ235, however, requires further investigation to ensure the basic glycolytic pathway is unaffected.

Grade 3–4 adverse effects associated with NVP-BEZ235 were reported by Carlo and colleagues^33^ in 50% of patients (5/10), without observing objective responses in the study group. A Phase Ib study of NVP-BEZ235, a dual inhibitor of PI3K and mTOR, was conducted in patients with advanced renal cell carcinoma. Most of the toxicity-related incidences, including fatigue, diarrhea, nausea, and mucositis, have been described as dose limiting. Thus, the finding that a combination of PI3K and mTOR blockade resulted in a high frequency of adverse events is not unexpected^34^. In in vivo studies, mice could survive well after adjusting to the NVP-BEZ235 dose. The tumor volume was also reduced successfully in these animals; however, their body weight should be carefully monitored.

Hypopharyngeal cancer often involves the larynx and requires oncologic management; however, most cases are diagnosed at more advanced tumor stages and have a higher incidence of neck nodal metastasis. According to the treatment guideline, hypopharyngeal cancers are suggested to be treated with total laryngectomy followed by postoperative radiotherapy (RT)^35^. To preserve the larynx among patients with advanced hypopharyngeal cancer, the combination of targeted therapy and RT compared with the use of RT alone was reported to be encouraging^36^. Molecular targeting agents have the potential to increase laryngeal preservation. Compared with other HNSCCs, HSCC needs more powerful targeting agents for laryngeal preservation. Therefore, NVP-BEZ235, cetuximab, and other targeting agents warrant further investigation.

This study has a limitation. Because injecting tumor cells into the hypopharynx is difficult, we injected the xenograft tumor into the flanks of mice in the in vivo study, which is not an appropriate site for FaDu cell growth.

In conclusion, NVP-BEZ235 alone could inhibit the proliferation and induce the apoptosis of FaDu cells. When NVP-BEZ235 was combined with Cisplatin, the two could synergistically inhibit the proliferation of FaDu cells by inducing cell cycle arrest in the G2/M phase through deregulating the PI3K-Akt-mTOR pathway; therefore, they could inhibit proliferation and induce apoptosis more effectively. Our study results suggest that NVP-BEZ235 could effectively sensitize Cisplatin-induced proliferation inhibition and apoptosis. The study results further confirm the effect of NVP-BEZ235 on FaDu cells and provide evidence for the clinical application of a combination of NVP-BEZ235 and Cisplatin in the treatment of hypopharyngeal cancer.

## Materials and methods

### Patients and samples

This study enrolled 12 male patients, aged 41–68 years (mean ± standard deviation, 52.3 ± 9.5), diagnosed with HSCC undergoing surgery at the Department of Otolaryngology, Kaohsiung Chang Gung Memorial Hospital, from 2009 to 2012. Clinical pathologic characteristics, including patients’ age, sex, TNM (Tumor, lymph node, metastasis) staging, and survival are presented in Table 1. Tumor samples and the adjacent noncancerous tissues were obtained immediately after resection and snap frozen in liquid nitrogen and stored until RNA extraction. Prior to tissue acquisition, informed consent was obtained from all patients. This study was approved by the Institutional Review Board of the Kaohsiung Chang Gung Memorial Hospital (IRB No. 100-4455A3).

**Table 1:** Characteristics of patients with HSCC

Characteristic	No. of patients
Sex	
Male	12
Female	0
Median age, y (range)	50.0 (41–68)
Staging	
I	0
II	4
III	0
IV	8
T stage	
T0	1
T1	0
T2	5
T3	2
T4a	4
T4b	0
N stage	
N0	5
N1	2
N2a	0
N2b	4
N2c	1
Two-year survival	
Expired	6
Survived	6

### qRT-PCR analysis of mTOR expression

The total RNA of cancerous and noncancerous tissues was obtained from patients with HSCC and from FaDu cells by using TRIzol reagent (Invitrogen; Life Technologies, Carlsbad, CA, USA). Complementary DNA (cDNA) was synthesized using a high-capacity cDNA reverse transcription kit (Applied Biosystems, Foster City, CA, USA), according to the manufacturer’s instructions. The 10-μl reaction volume contained 25 ng cDNA, 0.5 μl mTOR gene expression assay (Hs00234508_ml, Applied Biosystems) and 5 μl 2 × TaqMan Universal PCR Master Mix (Applied Biosystems) and was run in an ABI 7500 Fast Real-Time System (Applied Biosystems). The thermal cycling parameters of PCR were 95 °C for 10 min followed by 40 cycles of 95 °C for 20 s and 60 °C for 1 min. The expression level of the *mTOR* gene was normalized to the internal control *ACTB* to obtain the relative threshold cycle (Δ*Ct*). The relative expression in cancerous tissue compared with noncancerous tissue (ΔΔ*Ct*) is calculated as follows: ΔΔ*Ct* = [Δ*Ct* (cancerous tissue – *ACTB*) – Δ*Ct* (noncancerous tissue – *ACTB*)]. The fold change in cancerous tissue compared with untreated noncancerous tissue was then calculated using the 2^−ΔΔ*Ct*^ method.

### Cell culture

Human FaDu HSCC cells used in this study were purchased from the Food Industry Research and Development Institute, Hsinchu, Taiwan. The cells were maintained in minimum essential medium (MEM; Invitrogen) containing 10% fetal bovine serum and grown at 37 °C with 5% CO_2_.

### MTT assay

FaDu cells were treated with various concentrations of NVP-BEZ235 or Cisplatin or phosphate-buffered saline (PBS; as control) and the percentages of metabolically active cells were determined based on mitochondrial conversion of MTT to formazan. The assay included the following steps: 5000 cells in 100 μl subjected to different treatments were plated in triplicates in a 96-well plate and culture media were replaced with MEM (without phenol) containing 0.02% MTT (Sigma-Aldrich, St. Louis, MO, USA) and incubated for 4 h. The medium was then replaced with 200 μl dimethyl sulfoxide per well. The absorbance was read at 570 nm using a 96-well format DTX880 Multimode Detector (Beckman Coulter, Brea, CA, USA). The background absorbance was measured in wells containing only the dye solution and culture medium. Data presented were the absorbance values subtracted by the background absorbance values and the mean of the triplicates.

### Flow cytometric analysis

Flow cytometric analysis of stained cells was performed on a Beckman Coulter flow cytometer (Beckman Coulter, Fullerton, CA, USA). Dual staining of cells with Annexin V and PI was used to assess the percentages of apoptotic cells. Cells (1 × 10^6^) were washed in cold PBS and resuspended in 200 μl staining solution [10 mM 4-(2-hydroxyethyl)- 1-piperazineethane- sulfonic acid (HEPES) pH 7.4, 140 mM NaOH, and 2.5 mM CaCl_2_] containing 5 μl of Annexin V-FITC and 10 μ of 20 mg/ml PI (BD Pharmingen, Franklin Lakes, NJ, USA) for 15 min at room temperature in the dark. Percentages of viable cells (double negative for Annexin V and PI), early apoptotic cells (Annexin V-positive and PI-negative cells), and late apoptotic cells (double positive for Annexin V and PI) were calculated. For cell cycle analysis, cells (5 × 10^5^) were washed in PBS, fixed with cold 70% ethanol, and incubated with 25 μg RNAse A (Sigma-Aldrich) for 1 h at 37 °C. Prior to analysis, 25 μl (5 mg/ml) of PI (Sigma-Aldrich) was added and the percentages of the cell population in sub-G1, G1, S, or G2/M phases were calculated from histograms.

### Western blotting

Samples were extracted in radioimmunoprecipitation assay (RIPA) buffer [20 mM Tris-HCl pH 7.5, 150 mM NaCl, 1 mM disodium ethylenediaminetetraacetate dihydrate (Na_2_EDTA), 1 mM ethylene glycol-bis(β-aminoethyl ether)-*N*,*N*,*N*′,*N*′-tetraacetic acid (EGTA), 1% NP-40, 1% sodium deoxycholate, 2.5 mM sodium pyrophosphate, 1 mM β-glycerophosphate, 1 mM Na_3_VO_4_, and 1 μg/ml leupeptin]. For Western blotting, 30 μg of total lysates were separated by 8–15% sodium dodecyl sulfate-polyacrylamide gel electrophoresis and transferred to polyvinylidene difluoride membrane (Millipore, Darmstadt, Germany). After blocking with nonfat dry milk for 1 h, the membranes were incubated overnight with primary antibodies at 1:1000 dilutions. Primary antibodies and antibodies against phosphorylated epitopes used in this study were mTOR (Cell Signaling Technologies, Danvers, MA, USA), phospho-mTOR (p-mTOR; Ser2448) (Cell Signaling), p70S6K (Sigma-Aldrich), phospho-p70S6K (p-p70S6K; Thr389) (Cell Signaling), caspase 3 (Cell Signaling), cleaved caspase 3 (Cell Signaling), poly (ADP-ribose) polymerase (PARP), (Cell Signaling) and cleaved PARP (Cell Signaling). β-actin (1:5000 dilution; Sigma-Aldrich) was used as internal control. Secondary antibodies were horseradish peroxidase (HRP)-conjugated goat antimouse immunoglobulin G (IgG; Sigma-Aldrich) and goat anti-rabbit IgG (Sigma-Aldrich). The membranes were briefly incubated with Western Lightning Plus-ECL enhanced chemiluminescence substrate (PerkinElmer, Waltham, MA) to visualize the proteins.

### Xenograft studies

Male BALB/cAnN.Cg nude mice (age: 4–6 weeks; wt: 18–20 g) were purchased from the National Laboratory Animal Breeding and Research Center, Taipei, Taiwan. The mouse xenograft experimental procedures conducted in this study were approved by The Institutional Animal Care and Use Committee of Kaohsiung Chang Gung Memorial Hospital (IACUC No. 2014121610). BALB/cAnN.Cg nude mice were housed in pathogen-free conditions and experimental procedures were performed in accordance with the Guide for the Care and Use of Laboratory Animals under the supervision of authorized investigators. FaDu cells (5 × 10^6^) were inoculated subcutaneously into the back of nude mice under 2–4% isoflurane inhalational anesthesia. Tumors were measured in length and width using calipers every 2–3 days for 3 weeks. Mice were assigned randomly to receive NVP-BEZ235 (oral administration: 50 mg/kg/day for 3 weeks), Cisplatin (intraperitoneal injections: 3.5 mg/kg/day twice a week for 3 weeks), a combination of the two at the same dose and schedule, or placebo for negative control. Tumor material was harvested for immunohistological studies by formalin fixation.

### Immunohistochemical staining

Polyclonal antibodies against p70S6K (Sigma-Aldrich) and phospho-p70S6K (Cell Signaling) were used as the primary antibodies. The tumor sections were incubated with primary antibodies (1:100 dilutions) for 1 h and then incubated with biotinylated goat anti-rabbit antibodies for 30 min. An HRP-diaminobenzidine staining kit (Sigma-Aldrich) was used to visualize the specific binding of the secondary antibody to the primary antibody and the stained sections were examined using a Zeiss microscope (Zeiss, Gottingen, Germany).

### Statistical analysis

The data sets of MTT assay, apoptotic cell detection, cell cycle analysis, and tumor volumes of mice included at least three biological replicates, and the data were expressed as the mean ± standard deviation. For qRT-PCR, the values of Δ*Ct* were used for the statistical analysis of gene expression. A two-sample *t* test was used to determine statistical significance and null hypotheses of no difference were rejected if *p* values were <.05. SPSS (version 15.0; SPSS, Chicago, IL, USA) was used for all statistical analyses.
